# Sensory Sub- and Suprathreshold TENS Exhibit No Immediate Effect on Postural Steadiness in Older Adults with No Balance Impairments

**DOI:** 10.1155/2020/2451291

**Published:** 2020-02-28

**Authors:** Darja Rugelj, Marko Vidovič, Renata Vauhnik

**Affiliations:** ^1^University of Ljubljana, Faculty of Health Sciences, Biomechanical Laboratory, Ljubljana, Slovenia; ^2^University Rehabilitation Institute, Republic of Slovenia, Ljubljana, Slovenia; ^3^Arthron Institute for Joint and Sports Injuries, Celje, Slovenia

## Abstract

Transcutaneous electrical nerve stimulation (TENS) has been reported to attenuate postural sway; however, the results are inconclusive, with some indicating the effect and others not. The study aimed to evaluate the effect of sensory sub- and suprathreshold low-frequency TENS applied through the plantar surface and posterior aspect of shanks on postural sway. In a group of healthy community-dwelling older adults, TENS was delivered with two different current intensities: (1) subsensory which is below conscious perception and (2) suprasensory threshold which is within the range of conscious perception. Frequencies of the TENS stimulation were sweeping from 5 to 180 Hz and were delivered through the plantar surface and posterior shanks of both legs. Postural sway was measured with a force platform in eyes-open and eyes-closed conditions. To evaluate potential fast adaptability to TENS stimuli, the results were evaluated in two time intervals: 30 seconds and 60 seconds. The results indicated that TENS with the chosen frequencies and electrode placement did not affect postural sway in both the sub- and suprathreshold intensities of TENS, in eyes-open and eyes-closed conditions, and in 30-second and 60-second time intervals. In conclusion, given that in this study sub- and suprathreshold TENS applied via the plantar surface of the feet did not attenuate postural sway, it would be easy to conclude that this type of electrical stimuli is ineffective and no further research is required. We must caution against this, given the specificity of the electrode placements. We recommend that future research be performed consisting of individuals with balance impairments and with different positions of electrodes.

## 1. Introduction

Falls and their consequences for elderly community-dwelling adults are of increasing concern due to the increasingly elderly population. Finding successful interventions to prevent falls in older adults is crucial. Numerous strategies that can lead to decreased risk for falls and can decrease the incidence of falls exist [1, 2]. Increased postural stability and balance efficacy are among the most critical factors that have the potential for fall prevention [3]. The regulation of human postural control depends on the accurate processing of visual, vestibular, and various somatosensory stimuli [4, 5]. During standing and purposeful movements, people constantly and subconsciously use this information for postural correction or ongoing movements. Due to aging, pathology, or external interference, certain augmentation of somatosensory input when standing is often needed. Additional somatosensory stimuli from skin mechanoreceptors are believed to enhance the signals coming from muscle and joint proprioceptors, thus enabling better perception and integration of stimuli into the ongoing movement [5]. Different methods that specifically enhance somatosensory flow exist; among them, light touch by a fingertip of a stationary [6] or movable surface [5] or even self-touching [7] has been shown to decrease postural sway in elderly as well as in young persons [6]. Other types of sensory stimulations that were used for the enhancement of sensory information are stochastic resonance stimulation, textured material stimulation, and garment stimulation usually applied over lower limbs. They all exhibit a stabilizing effect on postural sway resulting in the decrease of the center of pressure (CoP) path length or its mean velocity [8].

Stochastic resonance stimulation has in recent years received increasing attention. It can be delivered with either imperceptible subthreshold vibratory stimuli delivered directly on the plantar surface [9] or subthreshold electrical stimuli [10–12] delivered via cutaneous sensory nerves. These imperceptible stimuli are believed to increase the noise in the central nervous system [10]. It is assumed that noise applied as imperceptible subthreshold vibratory or electrical stimuli attenuates feedback from the lower limbs. The proposed mechanism assumes that noise enables faster movement detection by skin receptors in the plantar surface or at the ankle joint in response to the postural sway and leads to a better perception of somatosensory stimuli [8]. The strength of the signal, which is interpreted as noise, is below conscious perception; therefore, subjects are not aware of its presence. This kind of stimuli decreases vibratory threshold detection in young [13] and elderly persons [14], enhances kinesthetic perception [15] and joint position sense [10], and decreases postural sway when skin receptors in the area above the muscles that stabilize posture are stimulated [11, 12, 15, 16].

The application of subthreshold electrical stimulation (ES) was reported to reduce postural sway in young healthy [11, 17] and healthy elderly adults [15, 18] and may consequently reduce the risk of falls in elderly persons. The reported studies differ in various features and protocols. Different types of electrical stimuli were used: stochastic resonance stimuli, white noise, and transcutaneous electrical nerve stimulation (TENS). Some researchers applied stochastic resonance [11, 12, 15], quasi-white noise with a sweep of frequencies from 5 to 1500 or 2000 Hz, and reported the decrease of postural sway [17]. A low-frequency conventional TENS was also used to attenuate postural sway. The frequency ranged from 100 to 200 Hz [16, 19, 20] with a threshold as well as suprathreshold stimulation with visible muscle contraction [21]. The parameters of electrical stimulation used in the studies largely differ. It is evident that additional studies are needed to determine the appropriate method and stimulation settings for rehabilitation purposes and for fall prevention programs of the elderly.

The devices that could deliver stochastic resonance electrical signals are not yet easily clinically available, while TENS devices are easily widely available and are routinely used in physiotherapy. TENS can modulate the response of the nervous system and enhance the functional state of elderly adults [22]. Since it may increase postural stability, it has the potential to decrease the tendency for falls in the elderly. It is thus necessary to investigate whether the stimuli in the form of a conventional TENS could attenuate postural sway similarly to stochastic ones and to find the proper combination of stimulation parameters and electrode placements for its optimal influence on postural control mechanisms. TENS is usually composed of short pulses (50–200 *μ*s) administered with a fixed or varying frequency in the range of 50 to 200 Hz.

To date, few investigators have studied the effect of conventional TENS on postural sway, and the results are inconclusive. For instance, Dickstein et al. [16] and Laufer and Dickstein [19] used conventional TENS with a constant pulse frequency of 100 Hz and suprathreshold intensities in a group of young healthy persons. The electrodes were placed above the gastrocnemius muscle [16] as well as in the posterior knee region [19]. In the case of electrode placement above gastrocnemius muscle, their results indicate a significant decrease in the average sway velocity as well as a trend toward a significant decrease in maximal mediolateral and anteroposterior sway velocity [16]. When the electrodes were applied on the medial and lateral aspects of the knees [19], a significant decrease in mean sway velocity was reported. In another study, Saadat et al. [20] found no effect of TENS stimulation, with the same frequency and intensity range, on the knee region in a group of diabetic neuropathy patients.

The intensities of the added somatosensory stimuli are important for the modulation of neuronal responses [23]. For instance, Breen et al. [13] reported the existence of an optimal level of stimulus intensity at which the subjects demonstrated the highest percentage of the improvement of vibratory threshold perception. The previous research with vibratory stimulation as well as electrical stimulation with white noise or stochastic stimuli used subthreshold stimuli intensity [11, 12, 15, 17] while TENS studies [16, 19, 20] used suprathreshold stimuli intensity.

The inconclusive results of the previous reports of TENS on the postural sway and repeated reports of vibratory stimuli via the plantar surface of the feet led to the purpose of the present work, which is to evaluate the effect of sensory subthreshold and suprathreshold TENS applied directly on the plantar surface of the feet, i.e., on the skin of metatarsal heads region and on the posterior aspect of the shanks below the knee, on postural sway in a group of healthy balance trained elderly adults. We hypothesized that TENS applied directly on the skin where the pressure fluctuations during postural sway are detected during standing would attenuate postural sway. The rationale for it would be in the fact that the electrical noise can facilitate the perception of tactile stimuli as has been already observed in older adults [14]. Furthermore, we hypothesized that suprathreshold TENS would attenuate postural sway to a greater extent as compared to subthreshold TENS. We also hypothesized that, in the eyes-closed conditions, the sub- and suprathreshold TENS would attenuate postural sway to a greater extent as compared to the eyes-open conditions.

## 2. Methods

### 2.1. Participants

Twenty-three healthy community-dwelling elderly adults participated in the study: 20 in the suprathreshold group (69.9 ± 7 years) and 19 in the subthreshold group (70.8 ± 6.2 years). The two experiments have been performed one following the other and the majority of participants were enrolled in both experiments. Their anthropometric data are presented in Table 1. The inclusion criteria were no prior lower leg injuries or conditions that could affect their balance, while the exclusion criterion was insensitivity to the pressure of the foot. All the participants experienced balance training of at least one year and did not exhibit balance impairment assessed with Mini-BESTest [24]. The study was approved by the Slovenian National Medical Ethics Committee (0120-309/2018/3) and, prior to any measurements, all participants read the information about the testing protocol, received additional verbal explanations when required, and provided written informed consent.

### 2.2. Procedure

#### 2.2.1. Sensory Threshold Testing

To ensure that electrical stimulation could be delivered via sensory cutaneous afferents, we measured the sensitivity of the skin on the soles. The sensory threshold of the soles was measured with Semmes-Weinstein monofilaments (Baseline Tactile Sensory evaluator, USA) ranging from 0.007 g to 300 g on a separate occasion before the application of TENS and measurements of postural sway. The sensory threshold testing is a valid and reliable procedure for evaluating the cutaneous threshold sensitivity [25, 26]. The test sites were chosen to correspond to the pressure distribution of the foot during standing. On both legs, three points on the soles were tested: beneath the first and the fifth metatarsal and on the heel. The initial leg was randomly chosen as well as the order of the testing points on the soles. Any areas with excessive callus were avoided. To determine the sensory threshold, a stepping algorithm was used according to the testing protocol described by Snyder et al. [26]. The examiner began by applying the 4.31 filament (2 g pressure). The result was considered positive when two of the three trials were correctly recognized. Depending on the result, the examiner proceeded to the thinner 3.61 filament (0.4 g pressure) or the thicker 4.60 (4 g pressure) one. The procedure was repeated until the thinnest sensed filament for each site was determined. The median values of the pressure thresholds for the elderly participants are shown in Table 2. These results correspond to normative values reported for the age group [27].

#### 2.2.2. Transcutaneous Electrical Nerve Stimulation

For the TENS, a Sono Plus 6920 (Enraf Nonius, the Netherlands) device was used. The self-adhesive electrodes (9 × 5 cm Axelgaard PALS, Axelgaard Manufacturing Co. Ltd., CA, USA) were placed on the plantar surface of the soles over the distal heads of metatarsals (−) and posterior shanks below the knee (+) to directly stimulate the skin of the plantar surface and the posterior aspect of the shanks. The position of the electrodes was adjusted to depolarize the type I and II cutaneous afferents responsible for conveying the touch and pressure information to the central nervous system. The decision for the particular electrode placement was based on the previous research of vibratory subsensory stimulation that reported decreased postural sway in young and elderly [9, 28], as well as persons with diabetic polyneuropathy [28]. Before applying the electrodes, the skin of the soles and shanks was cleaned with alcohol to remove the old skin and to decrease the surface impedance. The stimulation parameters were set at 300 µs pulse duration, frequency range from 5 to 180 Hz in a 12-second ramp-up and 12-second ramp-down sweeping between the two sets of frequencies. The intensity of the TENS was gradually increased until the subjects reported sensation. The intensity was then slowly decreased until the subjects were unable to perceive it. After subjects changed their position from sitting to standing on the force plate, the sensation of TENS was again checked and adjusted if necessary to a sub- or suprathreshold value.

#### 2.2.3. Postural Sway Testing

The Kistler 9286AA (Winthertur, Switzerland) force platform was used for data acquisition, with the corresponding BioWare data acquisition software. The force platform was used to assess the center of pressure movement during quiet upright standing. Data acquisition lasted 60 seconds at the 200 Hz sampling rate. All the analyses were later done on a Linux server (Fedora 24) with a StabDat-V2.0 software [29], which is a web-based application that had been developed for stabilometric measurements and consists of system procedures and data analysis programs written in C, Fortran, and PHP. This application is freely available upon request.

Four sway parameters in the time domain were chosen for the analysis of postural sway: [1] CoP velocity [2], mediolateral [3] and anteroposterior path lengths [4], and sway area calculated as the best area outline represented by the first 20 Fourier coefficients (FAO) as described elsewhere [30]. The test-retest reliability of the used postural sway measurements on the force platform in open and closed eyes has been established for healthy elderly as excellent to very good (ICC from 0.68 to 0.85) [31]. For both experiments, postural sway variables were analyzed for two time series of 60 and 30 seconds.

Participants were instructed to stand barefoot, as still as possible, with their feet close together on the force platform. Arms were relaxed beside the body, while the head was held upright and looking forward to an anchor point at eye height approximately two meters away. The measuring procedure was immediately stopped if the participants opened their eyes or moved their feet or arms from the required position. Between the measurements, the participants were allowed to rest in a sitting position for at least 60 seconds.

A nonslip insulating rubber pad was installed on the force platform to avoid any electrical interference between the electrical stimulation and the force platform. To control for the potential interference, we have conducted a test with fixed frequencies of 60, 120, and 180 Hz. The potential interference was assessed by fast Fourier analysis of the ML and AP CoP displacement time series. For this purpose, a standard fast Fourier transformation (FFT) computer program was developed [32]. The resulting power spectra were plotted and visually inspected for the presence of the applied stimulation frequencies.

#### 2.2.4. Testing Protocol

The research procedure consisted of two experiments. The first one had a subthreshold TENS in eyes-open and eyes-closed conditions, and a randomized double-blind protocol of TENS delivery was used. The second experiment, with a suprathreshold TENS also with eyes open and closed, used a randomized protocol. A random number generator was used to determine the order of tests. The participants and the examiner were blinded for the presence or absence of the subthreshold TENS, while the electrical stimulation was operated by a research assistant. In each experiment, measurements of postural sway were performed under four different sensory conditions while the subject was standing on the force platform. The order of testing was randomized in two blocks: first with eyes open or closed and then in each vision condition the subsensory TENS or no TENS (subsensory experiment) and suprasensory TENS and no TENS (suprasensory experiment).

#### 2.2.5. Statistical Analysis

The Statistical Package for Social Sciences (SPSS 24, SPSS Inc., Chicago, IL USA) was used for the statistical analysis. A repeated measures analysis of variance (ANOVA) was performed to identify the effect of the vision conditions and TENS on four postural sway-dependent variables. Significant ANOVA findings were followed up by the paired sample *t*-test and Bonferroni post hoc tests. The significance level was set at *p* < 0.05.

## 3. Results

### 3.1. Subthreshold Stimulation

Data distribution was assessed for normality with the Shapiro-Wilk test. Since data showed normal distribution, the data were analyzed using parametric tests. A (2 × 2) repeated measures ANOVA was calculated comparing the postural sway variables each in four different experimental conditions, i.e., for the open and closed eyes conditions without and with subthreshold TENS. The analysis was performed for 60- and 30-second time series.

For the 60-second time series, a significant main effect for the vision condition was found for all the analyzed postural sway variables: mean CoP velocity (*F*_1_ = 60.01, *p* < 0.001; *η*^2^ =0.769), mediolateral path length (*F*_1_ = 62.014, *p* < 0.001; *η*^2^ = 0.0775), anteroposterior path length (*F*_1_ = 45.902, *p*=0.001; *η*^2^ = 0.718), and sway area (*F*_1_ = 24.842, *p*=0.001; *η*^2^ = 0.580), indicating the expected effect of vision on the postural sway variables.

The main effect of subthreshold TENS was not significant for all the analyzed postural sway variables: mean CoP velocity (*F*_1_ = 0.582, *p*=0.455; *η*^2^ = 0.769), mediolateral path length (*F*_1_ = 0.152, *p*=0.701; *η*^2^ = 0.008), anteroposterior path length (*F*_1_ = 2.507, *p*=0.131; *η*^2^ = 0.122), and sway area (*F*_1_ = 0.566, *p*=0.461; *η*^2^ = 0.030), indicating no effect of TENS on postural sway variables. The interaction between open or closed eyes and TENS conditions was also not significant for all the analyzed postural sway variables: mean CoP velocity (*F*_1,18_ = 0.226, *p*=0.640, *η*^2^ = 0.012), mediolateral path length (*F*_1,18_ = 0.126, *p*=0.726; *η*^2^ = 0.007), anteroposterior path length (*F*_1,18_ = 0.469, *p*=0.502; *η*^2^ = 0.025), and sway area (*F*_1,18_ = 0.225, *p*=0.641; *η*^2^ = 0.012), indicating that there was no different response to the TENS or no-TENS conditions. The detailed results for all reported time-domain CoP variables are given in Table 3.

We further conducted a post hoc pairwise comparison for eyes-open and eyes-closed conditions, comparing TENS and no-TENS conditions. The results indicate no significant differences for all postural sway variables in the eyes-open (*p* ranging from 0.344 to 0.941) and eyes-closed conditions (*p* ranging from 0.196 to 0.768). It can be seen that the values for all four sway variables are not significantly different during the TENS conditions with eyes open or closed compared to the no-TENS condition.

We further analyzed the first 30 seconds of time series for the experiment with subthreshold stimulation and used the same statistical methods as for the 60-second time series. A significant main effect for the vision condition was found for all the analyzed postural sway variables: mean CoP velocity (*F*_1_ = 56.57, *p* < 0.001; *η*^2^ = 0.759), mediolateral path length (*F*_1_ = 55.81, *p* < 0.001; *η*^2^ = 0.756), anteroposterior path length (*F*_1_ = 43.22, *p* < 0.001; *η*^2^ = 0.706), and sway area (*F*_1_ = 36.25, *p* < 0.001; *η*^2^ = 0.668), indicating an effect of vision on postural sway variables.

The main effect of TENS was not significant for all the analyzed postural sway variables: mean CoP velocity (*F*_1_ = 0.127, *p*=0.726; *η*^2^ = 0.007), mediolateral path length (*F*_1_ = 0.061, *p*=0.808; *η*^2^ = 0.003), anteroposterior path length (*F*_1_ = 2.285, *p*=0.148; *η*^2^ = 0.113), and sway area (*F*_1_ = 0.204, *p*=0.657; *η*^2^ = 0.011), indicating no effect of TENS on postural sway variables. The interaction between open or closed eyes and TENS conditions was also not significant for all the analyzed postural sway variables: mean CoP velocity (*F*_1,18_ = 0.485, *p*=0.495; *η*^2^ = 0.026), mediolateral path length (*F*_1,18_ = 0.426, *p*=0.0522; *η*^2^ = 0.023), anteroposterior path length (*F*_1,18_ = 0.397, *p*=0.537; *η*^2^ = 0.022), and sway area (*F*_1,18_ = 0.343, *p*=0.565; *η*^2^ = 0.019), indicating that there is no different response to the TENS or no-TENS conditions. The detailed results for all reported time-domain CoP variables are given in Table 3.

### 3.2. Suprathreshold TENS Stimulation

For the 60-second time series, a (2 × 2) repeated measures ANOVA was calculated, comparing the postural sway variables, each in four different experimental conditions, i.e., for the eyes-open and eyes-closed conditions without and with subthreshold TENS. A significant main effect for the vision condition was found for all the analyzed postural sway variables: mean CoP velocity (*F*_1_ = 106.80, *p* < 0.001; *η*^2^ = 0.856), mediolateral path length (*F*_1_ = 76.01, *p* < 0.001; *η*^2^ ^=^ 0.809), anteroposterior path length (*F*_1_ = 106.70, *p*=0.001; *η*^2^ = 0.856), and sway area (*F*_1_ = 96.58, *p*=0.001; *η*^2^ = 0.843), indicating an effect of vision on postural sway variables.

The main effect of TENS was not significant for all the analyzed postural sway variables: mean CoP velocity (*F*_1_ = 0.268, *p*=0.611; *η*^2^ = 0.015), mediolateral path length (*F*_1_ = 0.014, *p*=0.907; *η*^2^ = 0.001), anteroposterior path length (*F*_1_ = 1.143, *p*=0.299; *η*^2^ = 0.060), and sway area (*F*_1_ = 0.262, *p*=0.615; *η*^2^ = 0.014), indicating no effect of TENS on postural sway variables. The interaction between open or closed eyes and TENS conditions was also not significant for all the analyzed postural sway variables: mean CoP velocity (*F*_1,18_ = 2.038, *p*=0.611; *η*^2^ = 0.102), mediolateral path length (*F*_1,18_ = 1.109, *p*=0.306; *η*^2^ = 0.58), anteroposterior path length (*F*_1,18_ = 2.944, *p*=0.103; *η*^2^ = 0.141), and sway area (*F*_1,18_ = 2.658, *p*=0.120; *η*^2^ = 0.129), indicating that there is no different response to the TENS or no-TENS conditions. The detailed results for all reported time-domain CoP variables are given in Table 4.

We further conducted a post hoc pairwise comparison for eyes-open and eyes-closed conditions, comparing suprathreshold TENS and no-TENS conditions. The results indicate no significant differences for all postural sway variables in eyes-open (*p* ranging from 0.097 to 0.202) and in eyes-closed conditions (*p* ranging from 0.149 to 0.534). It can be seen that the values for all four sway variables are not significantly different during the TENS conditions with eyes open or closed compared to the no-TENS condition in the subthreshold experiment and in the suprathreshold experiment.

We further analyzed the first 30 seconds of the time series for the experiment with suprathreshold stimulation and used the same statistical methods as for the 60-second time series. A significant main effect for the vision condition was found for all the analyzed postural sway variables: mean CoP velocity (*F*_1_ = 105.14, *p* < 0.001; *η*^2^ = 0.854), mediolateral path length (*F*_1_ = 69.80, *p* < 0.001; *η*^2^ = 0.795), anteroposterior path length (*F*_1_ = 132.40, *p* < 0.001; *η*^2^ = 0.880), and sway area (*F*_1_ = 54.02, *p* < 0.001; *η*^2^ = 0.750), indicating an effect of vision on postural sway variables.

The main effect of TENS was not significant for all the analyzed postural sway variables: mean CoP velocity (*F*_1_ = 1.606, *p*=0.221; *η*^2^ = 0.082), mediolateral path length (*F*_1_ = 0.706, *p*=0.412; *η*^2^ = 0.038), anteroposterior path length (*F*_1_ = 3.284, *p*=0.087; *η*^2^ = 0.154), and sway area (*F*_1_ = 1.067, *p*=0.315; *η*^2^ = 0.056), indicating no effect of TENS on postural sway variables. The interaction between open or closed eyes and TENS conditions was also not significant for all the analyzed postural sway variables: mean CoP velocity (*F*_1,18_ = 2.685, *p*=0.119; *η*^2^ = 0.130), mediolateral path length (*F*_1,18_ = 1.908, *p*=0.184; *η*^2^ = 0.096), anteroposterior path length (*F*_1,18_ = 3.340, *p*=0.084; *η*^2^ = 0.156), and sway area (*F*_1,18_ = 4.192, *p*=0.055; *η*^2^ = 0.189), indicating that there is no different response to the TENS or no-TENS conditions. The detailed results for all reported time-domain CoP variables are given in Table 4.

## 4. Discussion

The alterations of the proprioceptive signal due to aging are likely to increase the risk of falls. Therefore, finding the procedure that would overcome the age-related decrease of cutaneous flow from the plantar surface and the proprioceptive flow from sway stabilizing muscles is an important topic in elderly health care. The purpose of the present work was to evaluate the effect of sub- and suprathreshold TENS applied through the plantar surface and posterior aspect of the shank on postural sway, to compare the response to sub- and suprathreshold TENS and to compare the response to TENS between eyes-open and eyes-closed conditions in a group of elderly adults with no balance impairments. Our results indicated that TENS with the chosen electrode positions and frequency range had no statistically significant effect on postural sway in sub- and in suprathreshold conditions in the elderly adults, analyzed in two time intervals (30 and 60 seconds).

The targeted tissues for the stimulation in our study were the cutaneous receptors of the plantar skin and shank arising from the same area where the sensory afferents that convey information of the center of pressure during unsupported standing are located. This so-called direct approach was described by Breen et al. [13] and resulted in an enhanced vibratory threshold for young and elderly persons [14]. Stochastic resonance was the most plausible explanation for the previously reported results [33]. We hypothesized that the stimulation applied directly over the target tissue (in our case, the skin of the plantar surface) could have changed the sensitivity to small fluctuations of CoP. The sensibility of the foot contributes to the regulation of the standing balance, especially in more demanding conditions, such as standing with eyes closed [34]. Therefore, decreased sensitivity of the plantar area was an exclusion criterion, and pressure sensitivity testing indicated that all participants had a normal pressure sensitivity for their age. Normative data for the foot [27] reported a gradual decrease of threshold sensitivity with age and it is 4 g for persons over 80 years of age. Therefore, in our group of community-dwelling elderly adults, the receptors and its axons were optimal for the age group and allowed for sub- and suprasensory stimuli to be transmitted to the central nervous system.

There are several features of the TENS, such as intensity, frequency settings, irregularity of the stimuli, and electrode position, which can contribute to the effect of TENS and offer possible explanations for the results obtained. To address the intensity question, we have conducted both the sub- (imperceivable stimulation) and suprathreshold (perceivable stimulation) TENS experiments. The three reported studies that used TENS as a stimulation mode [16, 19, 20], for instance, reported threshold TENS stimulation. Based on the present results, we can conclude that the stimulation intensity did not play any role in the effect of TENS on postural sway. The suprathreshold experiment allowed us to compare the obtained results with the previous TENS studies. Our results are in agreement with Saadat et al. [20], while Dickstein et al. [16] and Laufer and Dickstein [19] reported a mild decrease of postural sway as a result of threshold TENS.

TENS used as a pain control tool has a high placebo effect [35], and therefore, a sham stimulation and double-blind design are required to control for the placebo effect. In the subthreshold experiment, the participant and the examiner were blinded to the TENS condition (subthreshold TENS or no TENS). With this, we can exclude the placebo effect. Only one of the previous studies had a placebo group [20], and TENS had no effect on postural sway, whereas studies without a control group or sham TENS [16, 19] reported a mild effect on postural sway. In the first subthreshold TENS experiment, care was taken to apply subthreshold intensity. However, it is difficult to control the subthreshold intensity, and it could be because the intensity of the stimuli was too low to be able to attenuate the postural sway response. Breen et al. [13] reported an optimal level of stimulation intensity, whereby subjects demonstrated the highest percentage of the improvement of vibratory threshold perception. In contrast, in the suprathreshold experiment, both the participant and examiner were aware of the presence of the stimulation. The results indicate that neither sub- nor suprathreshold TENS modulated the postural response as compared to the no-stimulation conditions. As a result, the postural sway remained in the same range as in the no-stimulation conditions.

Additionally, we analyzed two time series of the postural sway data, one of 60 seconds and another of 30 seconds. This allowed us to assess the possible habituation effect of TENS stimuli [36] as well as to compare and discuss our results with regard to the previous reports. The results showed that the data acquisition time did not influence the postural response of the participants. We can conclude that neither the adaptation to TENS nor the fatigue influenced the obtained results. The time frame of 30 seconds also allowed us to compare our results to the previous TENS studies of Saadat et al. [20], Dicksten et al. [16], and Laufer and Dickstein [19], who all used 30-second time frames of data acquisition and found no [20] or limited effect of TENS on postural stability [16, 19]. Although the results of longer data acquisition for postural sway assessments have better reliability [37], the agreed minimal reliable time for data acquisition was 30 seconds [38].

Furthermore, the irregularity of the stimuli could play an essential role in the subthreshold stimulation. For TENS, it is known that the nervous system habituates to constant stimuli [36]. To avoid habituation to TENS stimuli, we introduced frequency modulation from 5 to 180 Hz in a 12-second ramp-up and 12-second ramp-down interval. The sweep of different frequencies (5–1000) was also used by Kimura and Kouzaki [17] to produce noise-like stimuli with much higher frequencies and resulted in a decrease in three of eight CoP variables. Studies in which stochastic resonance was used as a stimulation mode reported a decrease of postural sway [11, 12]. Toledo et al. [15] reported a decrease of postural sway only in more demanding conditions. In our study, the frequency sweep between 5 and 180 Hz was used to obtain irregularity of the stimuli, while Saadat et al. [20] reported applying a constant frequency of 100 Hz, as did Dickstein et al. [16] and Laufer and Dickstein [19]. Since there was no response in either experiment in our study and no response in the study of Saadat et al. [20], we might conclude that, regardless of regular or irregular frequency of stimuli, the frequencies in the range up to 200 Hz do not decrease the threshold for detection of pressure stimuli. This conclusion is also in agreement with Garsia et al. [23] who reported that none of the four frequencies (3, 30, 150, and 300 Hz) was superior for the modulation of corticospinal excitability.

Finally, the chosen electrode position could have influenced the results with a predominantly inhibitory influence on the spinal circuits, similarly as in the case of H-reflex modulation [39]. The cutaneous afferents of the sole of the foot can adjust the excitability of spinal motoneurons that innervate the muscles that act around the ankle joint [39]. There is an electrode position-dependent response of H-reflex, in which the electrodes positioned on the metatarsal area caused inhibition of H-reflex, and the electrodes positioned on the heel resulted in its facilitation [39]. In our experiments, the electrodes were placed on the metatarsal area of the plantar surface; however, the current loop was through the posterior shank where the afferents from the skin of the heel area could be influenced by electrical currents. Given the possible inhibitory influence of the applied TENS, which is the opposite of the stochastic resonance [33], the net result of these two processes may lead to no change between the experimental conditions. Additionally, it was also reported that balance training causes a reduction of H-reflex [40, 41]. Given that the elderly adults in our group had experienced balance training, it is thus also possible that their postural control mechanisms are optimal and might not be enhanced by the applied TENS. However, this assumption contradicts previously reported results. Dickstein et al. [16] and Laufer and Dickstein [19] performed their experiment with young able-bodied participants whose postural control mechanisms were also optimal. Additionally, the feet position in their experiments was 15 cm apart, which offered even greater postural stability. Therefore, we can exclude the age-optimal balance as a potential factor for no effect of TENS on postural sway. Specifically, regardless of training, the postural steadiness of older adults is still weaker as compared to that of young adults [31]. Hence, if TENS had a beneficial effect on young able-bodied persons with optimal postural control, it was correct to expect the same effect for healthy elderly persons.

In this study, every effort was made to obtain unbiased results. We controlled for (1) pressure testing, indicating that there was proper conductivity of the sensory stimuli from the area, (2) blindness to the presence of TENS in subthreshold experiment, in order to control for the placebo effect of TENS, (3) short (30 seconds) and long (60 seconds) acquisition periods in order to control for the adaptation to stimuli and control for the reliability of the stabilometric data, and (4) sweep of frequencies in order to control for possible adaptation to regular TENS stimuli. Therefore, the possible explanation for the obtained results could be in the combination of the electrode positions and chosen stimulation parameters. Both conditions are reported to influence the regulatory mechanisms in the spinal cord.

Given that in this study sub- and suprathreshold TENS applied via the plantar surface of the feet did not attenuate postural sway, it would be easy to conclude that this type of electrical stimulus is ineffective and that no further research is required. We caution against this given the specificity of the electrode placements, and possibly the population studied. We recommend that future research be performed that consists of individuals with balance impairments and with different positions of electrodes such as over gastrocnemius muscle or above tibialis anterior muscle coupled with varied intensity, frequency, and duration of TENS pulses. Namely, all of the abovementioned parameters of TENS can influence the quality and quantity of the response to the electrical stimulation [23]. Shorter pulse width would increase the selectivity, i.e., avoid depolarizing pain sensory fibers, and the intensity higher than the perceptual threshold would induce more modulation [23]. Promising results with suprathreshold TENS were reported for chronic stroke persons with mild to moderate balance impairment [42].

## 5. Conclusion

In conclusion, the influence of TENS on postural sway remains inconclusive. The basic assumption for the use of TENS-type of electrical stimulation for the facilitation of information processing was that regular or quasi-regular signals produced by low-frequency TENS delivered through plantar surface could alter the random nature of postural sway, i.e., the movement of the center of pressure, which is related to the activation of pressure receptors in the plantar surface. The electrical stimulation was expected to improve the processing of information, thus allowing the detection of weaker signals and responding accordingly. We can conclude that a combination of low frequencies and predictable sweeps of frequencies with sub- and suprathreshold intensities delivered through a plantar surface is not the type of stimulation to increase sensitivity to CoP fluctuations in response to postural sway in a group of elderly adults without balance impairments.

## Figures and Tables

**Table 1:** Descriptive data of the participants in the study (*N* = 23).

	Mean ± SD	Minimum	Maximum
Age (years)	70.8 ± 6.2	60	83
Body mass (kg)	65.4 ± 11.9	48	90
Body height (cm)	164.3 ± 6.6	155	182
BMI (kg/m^2^)	23.9 ± 3.1	18.3	31.1
Mini-BESTest (points)	25.5 ± 1.8	21	28

Mini-BESTest: Mini-Balance Evaluation System Test [24].

**Table 2:** Median values of the threshold pressure values for the group of elderly adults.

	1^st^ metatarsal		5^th^ metatarsal		Heel	
	Median (min–max)		Median (min–max)		Median (min–max)	
	Left	Right	Left	Right	Left	Right
Filament no.	4.6 (4.31–6.65)	4.6 (4.31–6.65)	4.6 (4.31–6.65)	4.6 (4.31–6.65)	4.31 (4.31–6.65)	5.62 (4.31–6.65)
Pressure (g)	4 (2–300)	4 (2–300)	4 (2–300)	4 (2–300)	2 (2–300)	30 (2–300)

**Table 3:** The mean values of four time-domain variables of postural sway for the experiment with subthreshold stimulation for 60 seconds and for the first 30 seconds of postural sway measurements.

	Mean velocity ± SD (cm/s)	ML path ± SD (cm)	AP path ± SD (cm)	Sway area ± SD (cm^2^)
60 seconds time series				
Open eyes	1.70 ± 0.51	73.46 ± 23.59	55.60 ± 17.28	5.60 ± 3.45
Open eyes with sub-TENS	1.67 ± 0.41	73.25 ± 21.07	53.18 ± 12.53	4.93 ± 1.83
Closed eyes	2.82 ± 0.98	122.17 ± 44.02	91.76 ± 34.36	9.10 ± 3.69
Closed eyes with sub-TENS	2.71 ± 1.03	118.82 ± 49.75	86.18 ± 31.26	8,86 ± 4.41
30 seconds time series				
Open eyes	1.80 ± 0.55	38.71 ± 12.30	29.64 ± 9.89	3.93 ± 2.73
Open eyes with sub-TENS	1.84 ± 0.51	41.10 ± 13.52	28.43 ± 7.21	3.50 ± 1.36
Closed eyes	3.09 ± 1.11	67.35 ± 24.46	90.05 ± 19.92	6.94 ± 2.93
Closed eyes with sub-TENS	2.99 ± 1.14	66.20 ± 28.64	47.04 ± 16.28	6.98 ± 3.18

ML: mediolateral; AP: anteroposterior; SD: standard deviation.

**Table 4:** The mean values for the 60-second and 30-second time series of the four postural sway variables in the suprathreshold stimulation experiment.

	Mean velocity ± SD (cm)	ML path ± SD (cm)	AP path ± SD (cm)	Sway area ± SD (cm^2^)
60 seconds time series				
Open eyes	1.57 ± 0.43	65.11 ± 23.71	54.54 ± 13.08	4.57 ± 2.07
Open eyes with TENS	1.49 ± 0.36	61.48 ± 18.12	52.00 ± 12.78	4.06 ± 1.68
Closed eyes	2.50 ± 0.54	102.22 ± 28.09	87.89 ± 19.47	7.82 ± 2.56
Closed eyes with TENS	2.67 ± 0.93	106.72 ± 43.98	95.82 ± 33.15	8.85 ± 3.54
30 seconds time series				
Open eyes	1.70 ± 0.48	35.48 ± 13.11	29.14 ± 7.76	3.40 ± 2.0
Open eyes with TENS	1.65 ± 0.44	34.44 ± 10.10	28.39 ± 7.84	2.85 ± 0.94
Closed eyes	2.82 ± 0.64	58.27 ± 17.12	48.74 ± 10.83	6.04 ± 2.79
Closed eyes with TENS	3.13 ± 1.13	63.66 ± 27.05	55.36 ± 18.71	7.63 ± 3.52

ML: mediolateral; AP: anteroposterior; SD: standard deviation.

## Data Availability

The data used to support the findings of this study are available from the corresponding author upon request.
